# P-481. Characterization of People with HIV Who Are Virologically Suppressed with Treatment Experience Using a US Real-World Database

**DOI:** 10.1093/ofid/ofae631.680

**Published:** 2025-01-29

**Authors:** Samir K Gupta, Katherine Cappell, Kwanza Price, Mac Bonafede, Joshua Gruber, Dylan Mezzio, Soodi Navadeh, Robert Sedgley, Sorana Segal-Maurer

**Affiliations:** Indiana University School of Medicine, Indianapolis, Indiana; Real World Evidence, Veradigm, Chicago, Illinois; Gilead Sciences, Inc., Foster City, California; Veradigm, Chicago, Illinois; Gilead Sciences, Forest City, California; Gilead Sciences Inc, Foster City, California; Gilead Sciences, Inc., Foster City, California; Real World Evidence, Veradigm, Chicago, Illinois; Division of Infectious Diseases, New York–Presbyterian Queens, Flushing, New York

## Abstract

**Background:**

People living with HIV who are virologically suppressed with treatment experience (PWH-VSTE) are often prescribed complex multi-tablet and/or multi-dose antiretroviral (ARV) regimens with increased risk of poor clinical outcomes due to adherence challenges, adverse events, and/or drug interactions. This population is not well characterized, with no universal definition. We conducted a retrospective, observational analysis of a large US database to characterize PWH-VSTE and better understand this subset of PWH.

**Figure:**
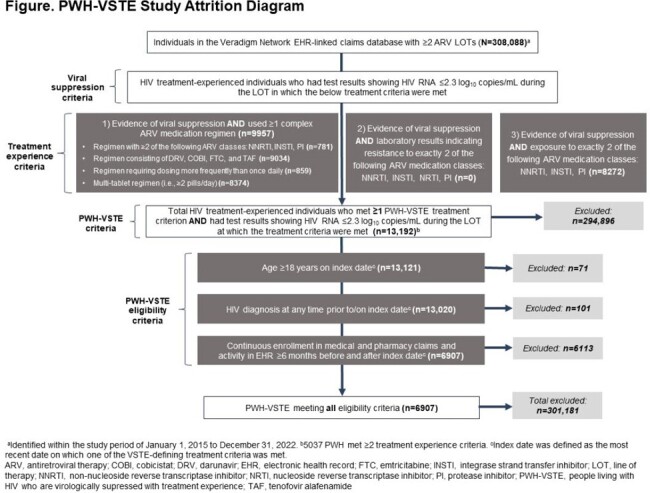

**Methods:**

PWH with treatment experience (PWH-TE) with ≥ 2 ARV lines of therapy (LOT) were identified from the Veradigm Network electronic health record (EHR) database linked with claims during the study period, Jan 2015–Dec 2022. We defined VSTE as virologic suppression (HIV-1 RNA ≤ 200 copies/mL) during the LOT in which ≥ 1 of the following treatment criteria were met: (1) used ≥ 1 “complex” ARV regimen; (2) resistance to exactly 2 ARV classes out of nucleoside or non-nucleoside reverse transcriptase inhibitors (NRTI, NNRTI), integrase strand transfer inhibitor (INSTI), and protease inhibitor (PI); (3) prior exposure to exactly 2 ARV classes out of NNRTI, INSTI, and PI **(Figure)**. For inclusion, PWH-VSTE were aged ≥ 18 years and had an HIV diagnosis, continuous claims enrollment, and EHR activity. We report the proportion of PWH-TE meeting VSTE-defining criteria, and their characteristics.

**Figure:**
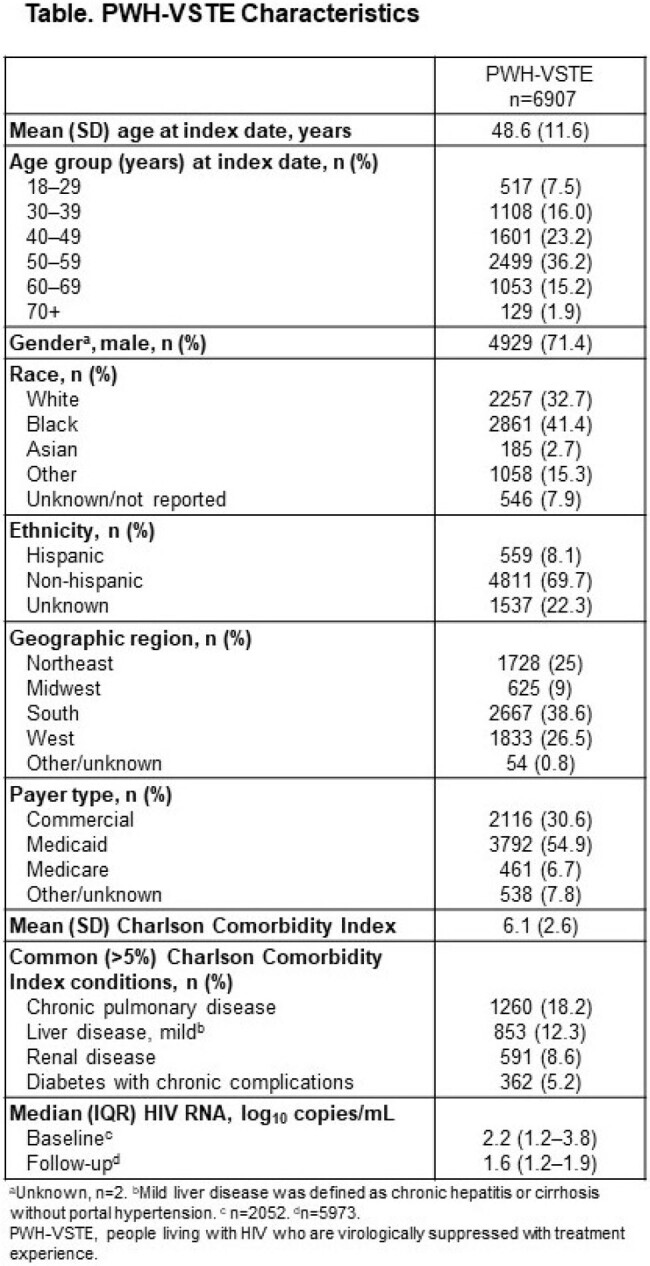

**Results:**

Of 308,088 PWH-TE, 13,192 (4%) met ≥ 1 VSTE-defining criterion (9957 met Criterion 1 and 8272 met Criterion 3); 6907 (52%) were eligible for analysis **(Figure)**. PWH-VSTE were mostly male (71%), Black (41%), aged 50–59 years (36%), using Medicaid (55%), from the South (39%), and had a mean (SD) Charlson Comorbidity Index of 6.1 (2.6) **(Table)**. Among those with data during the baseline period (6-month period prior to index date), 48.2% (990/2052) had a viral load of ≤ 200 copies/mL. Resistance testing was done for 13.3% of PWH-VSTE during the baseline period, but only 0.2% had results available.

**Conclusion:**

In this large, real-world database, PWH-VSTE comprised 4% of PWH-TE, were mostly male, and had a high level of comorbidities. Understanding the characteristics of PWH-VSTE will enable clinicians to better address the needs of this historically understudied population.

**Disclosures:**

**Samir K. Gupta, MD**, Gilead Sciences, Inc.: Advisor/Consultant|ViiV Healthcare: Advisor/Consultant|ViiV Healthcare: Grant/Research Support **Katherine Cappell, n/a**, Veradigm: Employee|Veradigm: Stocks/Bonds (Public Company) **Kwanza Price, n/a**, Gilead Sciences, Inc.: Stocks/Bonds (Public Company) **Mac Bonafede, PhD**, Moderna, Inc.: Advisor/Consultant|Veradigm: employees|Veradigm: Stocks/Bonds (Public Company) **Joshua Gruber, PhD MPH**, Gilead Sciences, Inc.: Employee|Gilead Sciences, Inc.: Stocks/Bonds (Public Company) **Dylan Mezzio, PharmD**, Gilead Sciences, Inc.: employees|Gilead Sciences, Inc.: Stocks/Bonds (Public Company) **Soodi Navadeh, PhD**, Gilead Sciences, Inc.: Employee|Gilead Sciences, Inc.: Stocks/Bonds (Public Company) **Robert Sedgley, n/a**, Veradigm: employee **Sorana Segal-Maurer, MD**, Gilead Sciences, Inc.: Advisor/Consultant|Gilead Sciences, Inc.: Grant/Research Support|Gilead Sciences, Inc.: Honoraria|Janssen: Advisor/Consultant|Theratechnologies: Advisor/Consultant|ViiV: Advisor/Consultant

